# A simple qualitative scale for diagnosis of laryngopharyngeal reflux: high correlations with pH measurements and disease severity. The usefulness of the Warsaw Scale in LPR diagnostics compared to other diagnostic tools

**DOI:** 10.1007/s00405-021-06989-x

**Published:** 2021-08-06

**Authors:** E. Wlodarczyk, A. Domeracka-Kolodziej, B. Miaskiewicz, H. Skarzynski, P. H. Skarzynski

**Affiliations:** 1 World Hearing Center, Institute of Physiology and Pathology of Hearing, Warsaw/Kajetany, Poland; 2 Institute of Sensory Organs, Kajetany, Poland; 3 Oto-Rhino-Laryngology Surgery Clinic, World Hearing Center, Institute of Physiology and Pathology of Hearing, Warsaw/Kajetany, Poland; 4 Heart Failure and Cardiac Rehabilitation Department, Faculty of Medicine, Medical University of Warsaw, Warsaw, Poland

**Keywords:** Laryngopharyngeal reflux (LPR), Warsaw Scale, Oropharyngeal pH monitoring

## Abstract

**Purpose:**

Diagnosis and monitoring of laryngopharyngeal reflux (LPR) is a constant challenge in otolaryngological practice, chiefly because there are no specific symptoms characteristic of the disease. In this paper, we present the validation of a simple, 6-level qualitative scale to gauge the clinical findings of LPR. It has been previously published in Polish as the Warsaw Scale.

**Methods:**

In the study, we enrolled 100 patients with voice problems who had registered in our clinic, and we performed an extended battery of diagnostic tests for LPR, together with 24-h pH monitoring.

**Results:**

The Warsaw Scale significantly outperformed other instruments in both predicting LPR status and correlating with pH measurements. Moreover, the rating provided by the scale showed a strong association with patient-reported symptoms.

**Conclusion:**

The data indicate that the Warsaw Scale could be used as an affordable, consistent, and effective diagnostic and monitoring tool for LPR.

## Introduction

Laryngopharyngeal reflux disease (LPR) was introduced as a medical term by the American Academy of Otolaryngology—Head and Neck Surgery in 2002 [1]. It describes a chronic condition involving a retrograde movement of fluid or gaseous contents of the stomach through the esophagus to the throat and larynx, causing acute or chronic symptoms of laryngitis. It is believed that damage to the larynx is mainly caused by the action of pepsin, not gastric acid [2]. As a result of this action, swelling of the laryngeal structures and metaplasia of the epithelium in the direction of the squamous epithelium occurs, the mucous glands overgrow, but then disappear. The most frequent symptoms of laryngeal mucosal damage include hoarseness, excess throat mucus, sensation of a foreign body, chronic cough, postnasal drip, and dysphagia [3].

Despite much work and many publications, controversies about the epidemiology, clinical presentation, diagnosis, and treatment persist. Different subjective and objective tools are used in diagnosing LPR, but there is no established standard procedure. A subjective and patient-oriented method is the Belafsky Reflux Symptoms Index [4]. Unfortunately, research has shown that factors such as sex and social or cultural conditions significantly affect its results [5]. Another patient and symptom-oriented questionnaire is the Voice Handicap Index (VHI). It was created to assess voice complaints and does not contain questions specific to the complaints observed in LPR. The Reflux Symptom Score (RSS), developed and proposed by Lechien, appears promising and useful. Although most of its patient-oriented and patient-reported outcome questions focus on laryngopharyngeal or voice symptoms, and do not take into consideration other, less specific symptoms, the RSS does contain questions about three primary groups of complaints: ear, nose, and throat disorders, abdominal disorders, and chest or respiratory disorders [6].

To assess whether LPR is present in patients, attempts have recently been made to create an easy questionnaire tool for physicians based on observations made in the laryngoscopy examination. Such tools include the Reflux Finding Score (RFS) by Belafsky [7] and the Reflux Sign Assessment (RSA) by Lechien [8]. However, the RFS does not account for some LPR symptoms such as leukoplakia, vocal fold erythema, posterior pharyngeal wall, and inflammation of the anterior pillars [9]. Moreover, it involves subjective evaluation of the signs without a clear definition of the rating [10]. The RSA, in turn, is a clinical instrument for evaluating laryngeal and extra-laryngeal findings associated with LPR.

In clinical practice, objective diagnostic methods are also used, such as pH monitoring, MII-pH monitoring, oropharyngeal pH monitoring, or the pepsin detection test. MII-pH monitoring detects esophageal bolus movement by measuring changes in electrical resistance. The usefulness of this method is limited by the fact that the number of reflux episodes may change from day to day, so that results can be false-positive or false-negative. Thus, some authors recommend using 28 h monitoring [11]. On the other hand, the results of pH monitoring may be unreliable if the proximal part of the sensor probe is allowed to dry [12]. Therefore, oropharyngeal pH monitoring was developed to measure pharyngeal pH with an antimony probe. With this method, measurements are possible both in a liquid or gaseous environment. Indeed, rigorous studies have shown that, compared to esophageal measurement, oropharyngeal pH measurement has higher positive predictive capability in LPR diagnosis [11]. A somewhat different approach, with promising results [13] are the pepsin detection methods that aim to detect pepsin in saliva. However, their reliability seems to depend on the measurement technique (immunoassay, ELISA, Western blot) and the reference threshold used to judge its outcome [14]. Additionally, a consensus regarding the optimum sampling time has not been yet reached [15].

Overall, there is no standardized method for diagnosing LPR. Each of the subjective and objective methods described above has its strengths and weaknesses. Moreover, it seems that, rather than clarifying, recent findings seem to complicate the problem [16]. For daily use, medical staff still need a tool that is easy to apply, inexpensive, and gives a reliable diagnosis.

One of the tools, based on years of clinical practice and experience, is the Warsaw Scale. It was proposed in 2014 as a simple and cheap diagnostic tool for identifying LPR. However, it was initially designed in Polish and was published in a Polish-language journal, resulting in a low level of awareness [17]. In this study, we validate its performance in predicting LPR in patients with voice problems. We comprehensively describe the scale in English and have generated an English translation of the questionnaire. We have then used it (in its Polish version) to evaluate the prevalence of LPR in our (Polish) clinical population, as assessed by 24 h pH monitoring. Finally, we have established the diagnostic power of the scale, correlated it with other widely used instruments (such as RSI, RFS, and VHI), and have calculated how well it predicts reflux severity.

## Materials and methods

### Participants

The study was performed in the Clinic of Audiology and Phoniatrics. We enrolled 100 patients who reported to the clinic with an undefined and undiagnosed voice disorder. Voice disorders included, for example, chronic or temporary hoarseness accompanied by other pharyngeal and laryngeal complaints. The mean age of the participants was 49.55 (SD: 13.84). There were more females (65%) than males (35%). All patients visited our clinic between January 2017 and June 2019. First, a medical interview seeking symptoms of laryngological, phoniatric, or general disease was undertaken. Then, diagnosis of LPR was approached using pH monitoring, two patient-filled questionnaires (the Reflux Symptom Index, RSI, and the Voice Handicap Index, VHI), and a simple 7-element internal questionnaire. The pharynx and the larynx were assessed using video laryngostroboscopy, and finally the Reflux Finding Score (RFS) and the Warsaw Scale was completed by an otolaryngologist.

### 24-h pharyngeal pH monitoring

The Restech^®^ pH sensor was calibrated in solutions of pH 7.0 and pH 4.0 before use. A catheter was inserted transnasally and advanced until a flashing LED was seen at the back of the subject’s throat; it was then positioned so that the flashing light was 5–10 mm below the uvula. The diameter of the LED light was 5 mm, and it served as a useful placement guide. The probe was secured to the patient’s face, as close to the nares as possible, using a Tegaderm^™^, and then passed over the ear and secured to the neck with a second Tegaderm^™^. The probe transmitter was taped to the skin or attached to the subject’s clothing with a clip-on case. A data recorder was attached to the patient’s belt. Patients were asked not to shower during the recording period and to keep a diary indicating meal times and the time spent horizontal (in bed) and vertical (out of bed). Meal times were excluded from the analysis of pharyngeal pH recordings (extending them by 5 min). Data from the Restech^®^ recorder were downloaded to a proprietary software program and correlated with the patient’s diary. The analysis involved counting the number of times (number of reflux episodes) that the pH dropped below 5.5 when upright or 5.0 when horizontal, as well as the duration of these events expressed as a percentage of the total monitoring time (the reflux time). LPR was diagnosed on the basis of a Ryan score, which is calculated as the percentage of time that the pharyngeal pH was below at the above mentioned threshold, the number of episodes over which the pH drops, and the duration of the drops (separately for vertical and horizontal body positions). According to the manufacturer, a Ryan score above 9.41 for the upright position and above 6.8 for the horizontal is considered abnormal [18, 19].

### Questionnaires

All questionnaires used in the study followed the original version as published by their authors: RSI and RFS by Belafsky [4, 7], VHI by Jacobson (Jacobson et al. 1997), and the Warsaw Scale by Domeracka-Kołodziej [17]. In addition, we also used an internal questionnaire with seven questions about strength of symptoms, frequency of symptoms, physical wellbeing, mood, social contact, everyday functioning, and general health. Participants were asked to answer each question using a numerical scale between 1 (low, bad, rarely) and 5 (high, good, often).

### Statistics

For a better presentation of Ryan scores, both horizontal and upright, we calculated the logarithm of the Ryan score, logRyan, according to the formula:

$$ {\text{logRyan}} = \ln \left( {{\text{Ryan}} + 1} \right), $$

where Ryan is the Ryan score of either the horizontal or upright position.

Computations were carried out in R. In hypothesis testing, it was assumed that a *p* value below 0.05 indicates rejection of the null hypothesis. Two-sample Student *t* tests or Welch’s one-way analysis of means were used to establish differences between two or more groups, respectively. The correlation was measured using the Spearman coefficient, while its significance was tested with Algorithm AS 89 [20]. The independence between two categorical variables was assessed by Fisher’s exact test.

To evaluate the performance of diagnostic tools in correctly predicting the status of LPR, the following metrics were used:

$$ {\text{Accuracy = }}\frac{{\text{TP + TN}}}{{\text{TP + TN + FP + FN}}}, $$

$$ {\text{Sensitivity = }}\frac{{{\text{TP}}}}{{\text{TP + FN}}}, $$

$$ {\text{Specificity = }}\frac{{{\text{TN}}}}{{\text{TN + FP}}}, $$

where TP is the number of true positives (i.e., the number of patients correctly diagnosed as those with LPR); TN is the number of true negatives (the number of patients correctly diagnosed as non-LPR); FP is the number of false positives (the number of patients mistakenly diagnosed as having LPR, although being healthy); and FN is the number of false negatives (the number of patients mistakenly diagnosed as being non-LPR, although being sick). The significance of the prediction, referred to as the no-information *p* value, is the probability that the observed accuracy could be achieved by guessing. Specifically, we calculated the one-sided binomial test with respect to no information.

All figures were prepared with R’s *ggplot2* package. In the case of box-plots, boxes indicate the 1st and 3rd quartiles, the horizontal line represents the median, and the vertical line represents the range between the lowest and highest value (unless the value exceeded the 1st or 3rd quartiles by more than 1.5 interquartile ranges).

## Results

The Warsaw Scale was created in answer to the need for a comprehensive laryngeal assessment in patients with GERD (confirmed in gastrological tests) who sought out a phoniatric specialist for help with their voice disorder. The main idea behind the Warsaw Scale is that a scale based on an image of the larynx might be less time-consuming and more comprehensive, making it useful in everyday clinical practice [17].

Below we show, step by step, the adequacy of the Warsaw Scale for diagnosis of LPR. The scale is first introduced and defined. Second, we show its predictive power for LPR identification. Next, we demonstrate its relationship to other widely accepted instruments. Lastly, we examine its correlation with objectively measured clinical presentation of LPR by pH monitoring.

### Warsaw Scale in English

For the purpose of this paper, we translated the original elements of the Warsaw Scale into English, as presented in Table 1. The Warsaw Scale is a six-level categorical scale with scores from the set {O,A,B,C,D,E}, where the order of the letters corresponds to increased severity of the disease. In contrast to other videolaryngoscopic-based tools, e.g., the RFS scale, the successive grades of the Warsaw Scale are interdependent, and a specific set of conditions must be fulfilled to achieve a given grade. The symptoms are evaluated in a binary manner–either they occur or they do not. For example, in RFS it is possible to have just two symptoms (of eight) with a score of 4, and have no other symptoms, to clear the pathological threshold of 7. However, in the Warsaw Scale, a clinically relevant link between observed pathological changes must be identified to establish higher grades of the disease. All patients in the current study were assessed according to the Polish-language version of the Warsaw Scale as published by Domeracka [17].

**Table 1:** Algorithm of the Warsaw Scale for diagnosing the severity of LPR

Definition of Warsaw Scale grades	
Grade	Prerequisites
O	No significant pathological changes in the larynx or pharynx
A	Symptom 1
B	Symptom 1 AND symptom 2
C	(symptom 1 or/and symptom 2) AND symptom 3
D	(symptom 1 or/and symptom 2 or/and symptom 3) AND symptom 4
E	(symptom 1 or/and symptom 2 or/and symptom 3 or/and symptom 4) AND symptom 5

Definition of symptoms	
Symptom identifier	Description of larynx and pharynx lesions
Symptom 1	Inflammation of the mucosa of the posterior pharyngeal commissure and posterior parts of vocal folds (nodules and ulcers)
Symptom 2	Inflammation of the mucosa of the arytenoid cartilage and the interarytenoid area
Symptom 3	Inflammation and hyperplasia of the area behind the arytenoid cartilage, vestibular folds, and aryepiglottic folds
Symptom 4	Inflammation and hyperplasia of the lower pharynx mucosa
Symptom 5	Oedemas of the infraglottis OR (dysfunction of crico-arytenoid joints) OR (contact granulomas) OR (overgrowth of vocal folds)

First, symptom groups are defined, which include lesions of the larynx and pharynx which are known outcomes of laryngopharyngeal reflux. Next, each grade is described as the co-occurrence of a set of pre-defined symptoms

### Description of population

In the study, we enrolled 100 patients, 65 females and 35 males, with mean ages of 49.2 and 50.1, respectively (Table 2). All were examined with four questionnaires: RSI, RFS, VHI, and the Warsaw Scale. The average RSI score was 20.02 (20.66 for females and 18.83 for males). The average RFS was found to be 6.62 (6.66 for females and 6.53 for males). The difference between the sexes was larger in the VHI scale – the average VHI for females was 22.48, whereas for males it was 16.8, for a grand average of 20.49. There were 19 cases of level O, 33 cases of level A, 37 cases of level B-level, 8 cases of level C, and 3 cases of level D. No cases of level E were observed in the current study.

**Table 2:** Statistics of the population based on scores reported in the LPR-diagnostic questionnaires

	All	Female	Male
*N*	100 (100%)	65 (65%)	35 (35%)
Age	Mean: 49.55 (SD: 13.84)	Mean: 49.23 (SD: 13.14)	Mean: 50.14 (SD: 15.22)
RSI	Mean: 20.02 (SD: 9.13)	Mean: 20.66 (SD: 9.04)	Mean: 18.83 (SD: 9.3)
RFS	Mean: 6.62 (SD: 2.8)	Mean: 6.66 (SD: 2.53)	Mean: 6.54 (SD: 3.28)
VHI	Mean: 20.49(SD: 20.29)	Mean: 22.48 (SD: 21.71)	Mean: 16.8 (SD: 17.04)
Warsaw Scale			
Warsaw Scale: O	19	12 (63.2%)	7 (36.8%)
Warsaw Scale: A	33	25 (75.8%)	8 (24.2%)
Warsaw Scale: B	37	20 (54.1%)	17 (45.9%)
Warsaw Scale: C	8	7 (87.5%)	1 (12.5%)
Warsaw Scale: D	3	1 (33.3%)	2 (66.7%)
Warsaw Scale: E	0	0	0

*SD* standard deviation

To assess the occurrence of LPR, 24 h pharyngeal pH monitoring was performed and the following metrics were obtained: number of reflux episodes, reflux time, and Ryan score, each of which were calculated for both the horizontal and upright positions (Table 3). In the whole population the average Ryan score in the upright position was 16.9, whereas the average Ryan score in the horizontal position was 1.21. Additionally, to facilitate clearer visualization and more reliable analysis, we applied a logarithmic transformation to obtain a logRyan score.

**Table 3:** Baseline measurements of 24 h pharyngeal pH monitoring

	All	Female	Male
Upright Ryan score	Mean: 16.9 (SD: 35.58)	Mean: 21.48 (SD: 42.69)	Mean: 8.39 (SD: 11.93)
Upright logRyan score	Mean: 1.7 (SD: 1.59)	Mean: 1.86 (SD: 1.68)	Mean: 1.41 (SD: 1.39)
Upright number of episodes	Mean: 4.54 (SD: 8.05)	Mean: 4.77 (SD: 7.74)	Mean: 4.11 (SD: 8.7)
Upright reflux time (%)	Mean: 1.13 (SD: 3.07)	Mean: 1.53 (SD: 3.71)	Mean: 0.39 (SD: 0.88)
Supine Ryan score	Mean: 1.21 (SD: 4.84)	Mean: 1.08 (SD: 3.98)	Mean: 1.43 (SD: 6.19)
Supine logRyan score	Mean: 0.27 (SD: 0.72)	Mean: 0.28 (SD: 0.71)	Mean: 0.25 (SD: 0.76)
Supine episodes	Mean: 1.11 (SD: 5.46)	Mean: 0.95 (SD: 4.09)	Mean: 1.4 (SD: 7.43)
Supine reflux time (%)	Mean: 0.89 (SD: 7.83)	Mean: 1.27 (SD: 9.69)	Mean: 0.18 (SD: 0.98)

*SD* standard deviation

According to studies on normative populations and the manufacturer’s guidelines, a Ryan score above 9.41 for the upright position or above 6.8 in the horizontal position is considered abnormal [18, 19]. Therefore, we qualified a patient to the LPR-positive group if one of the above conditions was fulfilled (Table 4). In total, 43 patients (43% of the total) were diagnosed as LPR-affected, among whom 30 were female (46%) and 13 were male (37%).

**Table 4:** Diagnosis of LPR based on 24 h pharyngeal pH monitoring

	All	Female	Male
Upright Ryan score > 9.41	41	29	12
Supine Ryan score > 6.8	5	3	2
LPR	43	30	13
No LPR	57	35	22

### Warsaw Scale effectively predicts the LPR diagnosis

To test the ability of the Warsaw Scale to diagnose LPR correctly, we compared the classification of patients based on 24 h pH monitoring with a prediction of disease using the Warsaw Scale. We assumed that scores “A” and “O” meant no LPR, while scores “B”,”C”, “D”, or “E” indicated the presence of LPR. This approach allowed us to correctly predict 46 True Negatives and 37 True Positives with 6 False Negatives and 11 False Positives (Table 5). Thus, the Warsaw Scale achieved a specificity of 80.7% and sensitivity of 86.0%, with a total accuracy of 83% (no-information rate test *p* value < 0.001).

**Table 5:** Comparison of LPR predictions from use of the Warsaw Scale and disease status as given by pH monitoring

(Number of patients)	Reference (pH monitoring)	
	No LPR	LPR
Prediction (Warsaw Scale)		
No LPR	46	6
LPR	11	37

Fisher’s test *p* value: $1.6 \cdot 10^{ - 11}$

To see how the Warsaw Scale performed relative to other known diagnostics tools, we established a protocol for differentiating between LPR-positive and LPR-negative patients using total scores from RSI, RFS, and VHI (Table 6). The best performing thresholds, in terms of accuracy, were: 21 for RSI, 9 for RFS, and 17 for VHI. That is, those tools reached accuracies of 68, 69, and 64%, respectively (no-information rate test *p* values: 0.016, 0.009, 0.094). Therefore, the accuracy of these three tools was at least 14% lower than the accuracy achieved with the Warsaw Scale (83%). Also, neither RSI, RFS, or VHI were able to surpass the Warsaw Scale in terms of sensitivity or specificity.

**Table 6:** Statistics of LPR prediction using different diagnostic instruments in comparison to diagnosis based on 24 h pH monitoring

Scale	Accuracy	Accuracy (*p* value)	Specificity	Sensitivity
Warsaw Scale	83.0%	**2.89·10**^**–8**^	80.7%	86.0%
RSI	68.0%	**0.016**	73.7%	60.5%
RFS	69.0%	**0.009**	75.4%	60.5%
VHI	64.0%	0.094	68.4%	58.1%
Symptom strength	60.0%	0.308	63.2%	55.8%
Symptom frequency	49.0%	0.956	43.9%	55.8%

Significance of accuracy was established using a one-sided binomial test with respect to the *no information rate*. In the case of the Warsaw Scale, all grades above A were treated as a positive LPR. For RSI, RFS, and VHI, the most predictive thresholds were chosen for each tool separately. For symptom strength and frequency, answers 3 or higher (out of 5) were taken to indicate LPR

Apart from the validated tools, patients were also asked to subjectively assess a) the strength and b) the frequency of symptoms they had using a simple numerical scale: from 1 (low, rare) to 5 (high, often). If this could successfully predict LPR, then its accuracy would be 60% using a perceived symptom strength of 3 as threshold (no-information rate test *p* value: 0.308) and using perceived symptom frequency of 49% as threshold (no-information rate test *p* value: 0.956). In conclusion, measurements from our population indicate that the Warsaw Scale is a promising tool for diagnosing LPR.

### Patient-oriented and clinical-based questionnaires correlate with the Warsaw Scale

**Fig. 1:**
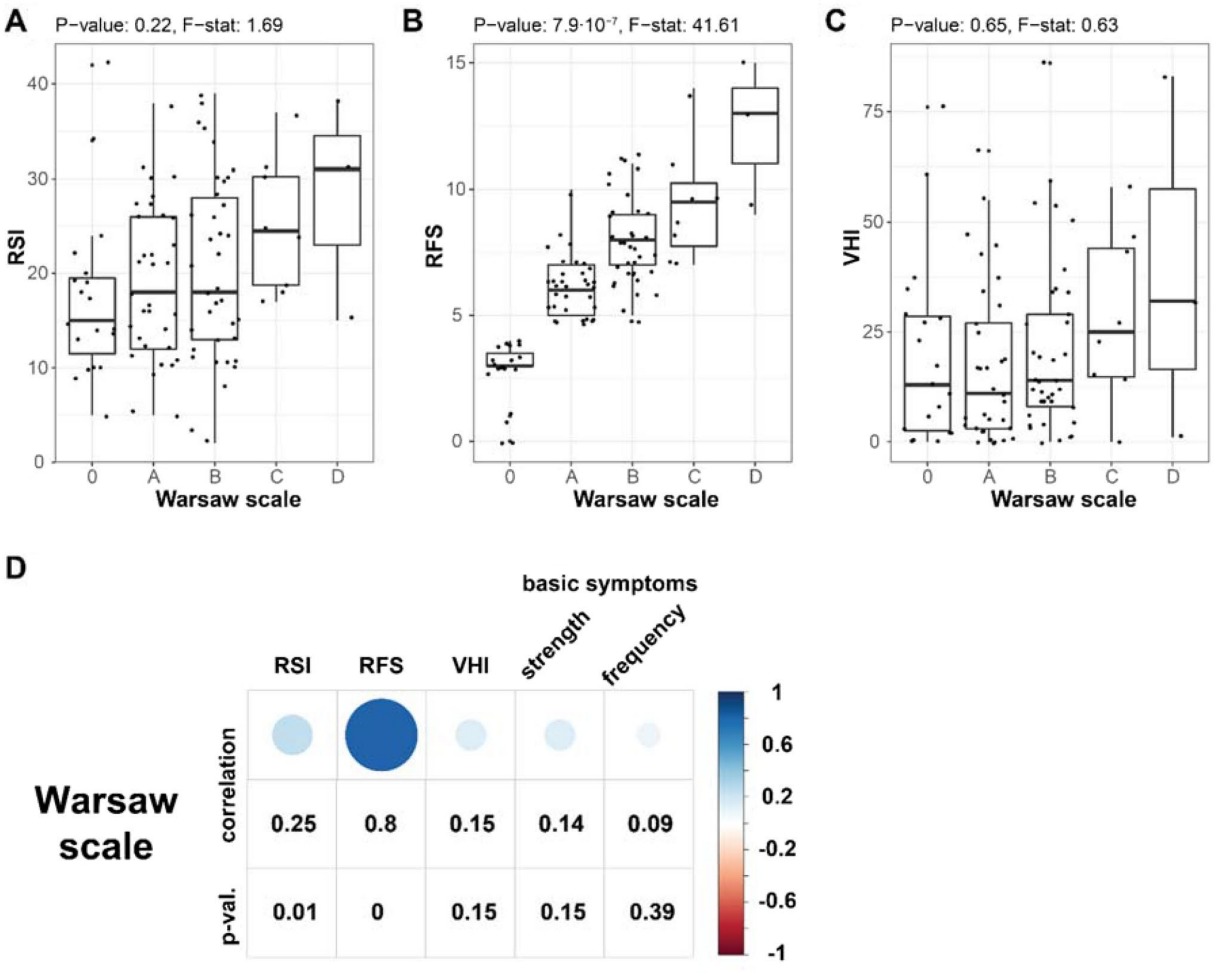
Association between the Warsaw Scale and other diagnostic instruments. **A**–**C** Comparison between distributions (depicted as box-plots) of the Warsaw Scale with **A** RSI; **B** RFS; and **C** VHI. **D** Correlation between Warsaw Scale levels and RSI, RFS, and VHI scores and symptom strength and frequency. One-way analysis of means was used to statistically test differences between groups. Spearman coefficient was used as a correlation metric. The *p *value of correlation is computed by a formal test for association between paired samples

To investigate further the relation between the Warsaw Scale and other diagnostic instruments, we performed a comprehensive analysis of association (Fig. 1). First, we compared how the values of other tools change with successive levels of the Warsaw Scale. As shown in Fig. 1A–C, the medians of RSI, RFS, and VHI did increase with increases in the Warsaw Scale grade. However, only the differences observed within RFS scores are statistically significant—shown using one-way analysis of means between several groups (Welch’s test *p* value = 7.9 10^–8^, *F* stat = 41.61). At the same time, differences within RSI (Welch’s test *p* value = 0.22, *F* stat = 1.69) and VHI (Welch’s test *p* value = 0.65, *F* stat = 0.63) are not statistically significant.

Second, the Warsaw Scale shows a positive correlation, calculated as the Spearman correlation coefficient, with all the tools (RSI, RFS, VHI, and basic symptoms) when considered as diagnostic instruments. However, a significant correlation could be observed only for comparison with RSI (correlation = 0.25, *p* value = 0.01) and RFS (correlation = 0.80, *p* value < 0.001). It should be noted that the correlation with RFS is especially high, which reflects the fact that both these scales are based on a videolaryngoscopic image and filled in by a physician. In conclusion, our data demonstrate that the Warsaw Scale measures a construct similar to that expressed by other widely accepted instruments.

### Severity of disease is associated with Warsaw Scale rating

As the last step of our considerations, we examined the relation of the Warsaw Scale to LPR pathology. This was done by comparing Warsaw Scale levels with objective measurements of the patients’ reflux episodes. The values obtained from 24 h pharyngeal pH monitoring of patients are directly linked to the severity of their disease. Thus, the correlation with pH measurements can be treated as a correlation with the stage and progression of LPR.

Specifically, we inspected (a) correlations between pH measures and the Warsaw Scale; (b) distributions of pH measures within successive levels of the Warsaw Scale. A positive association was found especially with respect to upright reflux measures (Table 6). Indeed, there was a high positive correlation (*r* = 0.70) between the Warsaw Scale and upright logRyan (*p* value = 3.6∙10^–16^). Similarly, there were also high correlations with the number of upright reflux episodes (0.59, *p* = 1.1∙10^–10^) and the upright reflux time (0.6, *p* = 2.0∙10^–11^). The Warsaw Scale clearly outperformed any other measure we administered(Table 7). The second-best diagnostic tool for our population, according to the correlation analysis, was RFS. Here, we obtained correlations of 0.6 (*p* = 4.70∙10^–11^) with upright logRyan, 0.44 (*p* = 5.40∙10^–6^) with upright number of episodes, 0.52 (*p* = 2.20∙10^–8^) with upright reflux time, and 0.31 (*p* = 1.70∙10^–3^) with horizontal logRyan.

**Table 7:** Correlation between 24 h pharyngeal pH monitoring measurements and the rating provided by different diagnostic instruments

	Upright						Supine	
	logRyan score		Number of episodes		Reflux time (%)		logRyan score	
	*r*	*p* value	*R*	*p* value	*r*	*p* value	*r*	*p* value
Warsaw Scale	0.70	3.6 10^–16^	0.59	1.1 10^–10^	0.61	2.0 10^–11^	0.19	0.062
RSI	0.35	3.2 10^–4^	0.35	4.3 10^–4^	0.33	8.10 10^–4^	− 0.02	0.86
RFS	0.6	4.7 10^–11^	0.44	5.4 10^–6^	0.52	2.20 10^–8^	0.31	1.70 10^–3^
VHI	0.17	0.092	0.13	0.18	0.2	0.041	0.08	0.46
Symptom strength	0.19	0.055	0.2	0.043	0.08	0.41	0.05	0.66
Symptom frequency	− 0.01	0.95	0	0.97	0.01	0.9	− 0.04	0.71

Spearman coefficient (*r*) was used as the correlation metric. The *p* value of the correlation was computed by a formal test for association between paired samples

**Fig. 2:**
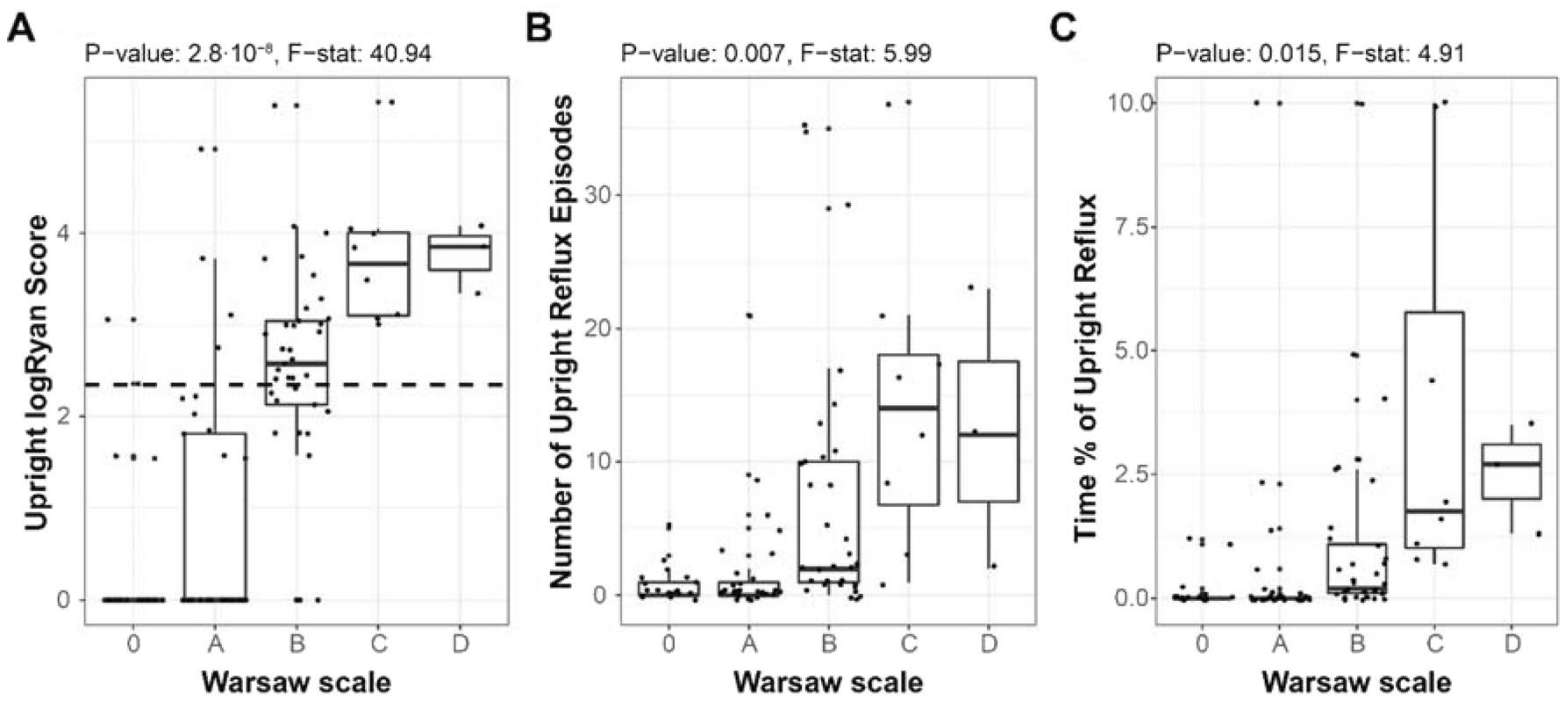
Distributions of upright 24 h pharyngeal pH measures according to Warsaw Scale level for **A** upright logRyan; **B** number of reflux episodes; **C** reflux time. Dashed line in panel **A** denotes the LPR-diagnostic threshold. Welch’s one-way analysis of means was used to statistically test differences between groups

Similarly, favorable behavior of the Warsaw Scale is seen when looking at how pH measures are distributed for different levels (Fig. 2). A formal comparison of means between groups demonstrates significant differences for all upright metrics: logRyan score (Welch’s test *p* value = 2.8∙10^–8^, *F* test = 40.94), number of reflux episodes (Welch’s test *p* value = 0.007, *F* test = 5.99), and reflux time (Welch’s test *p* value = 0.015, *F* test = 4.91).

Taken together, comparisons between the Warsaw Scale and pH monitoring suggest that successive grades of the Warsaw Scale strongly associate with more intense LPR.

## Discussion

The growing number of patients complaining of hoarseness, vocal fatigue, chronic throat clearing, postnasal drip, chronic cough, dysphagia, and globus makes one look for a simple diagnostic tool to recognize or exclude LPR. However, many of these symptoms are nonspecific and may be caused by other conditions such as smoking, allergies, and neurogenic mechanisms [21]. Findings of a laryngoscopy examination have slightly higher diagnostic value. Many authors argue that laryngeal edema and erythema are characteristic of LPR [22, 23].

It has already been demonstrated that 12% of patients with normal distal esophageal acid exposure have, according to dual-probe pH monitoring, abnormal proximal reflux [24]. To identify proximal reflux, a proximal probe needs to be positioned as high in the esophagus as possible; however, if the probe is placed too high there is the risk it will dry out and the pH measurement will become unreliable. The Restech pH monitoring device was developed for 24 h hypopharyngeal measurement, and its design allows the probe to be positioned in the pharynx above the upper esophageal sphincter. The probe’s unique teardrop shape prevents it drying out [25]. Studies that have simultaneously used a single pharyngeal probe and dual esophageal probes indicate that the pharyngeal probe can reliably capture reflux episodes that move proximally from the esophagus to the pharynx [11, 26]. Studies comparing pharyngeal and esophageal pH monitoring suggest that pharyngeal measurements have higher positive predictive capability than esophageal ones [27, 28]. However, performing both esophageal and pharyngeal pH tests is time-consuming and expensive. Therefore, questionnaires approximating the diagnosis of laryngopharyngeal reflux are useful clinical tools.

In our work, the Warsaw Scale was one of four questionnaires used (alongside RFS, RSI, and VHI). Two of them, VHI and RSI, are completed by the patient; the other two (the Warsaw Scale and RFS) are used by the specialist to systematically describe abnormalities seen in the larynx. Since all patients underwent 24 h pharyngeal pH measurement, we could look at the correlation between questionnaire answers and pH measures.

To facilitate the diagnosis of LPR, Belafsky proposed two questionnaires designed to measure the frequency and intensity of symptoms suggestive of laryngopharyngeal reflux. The first questionnaire was the Reflux Syndrome Index (RSI), completed by the patient, and the other was the Reflux Finding Score (RFS), completed by an otolaryngologist/phoniatric specialist based on the laryngoscopic presentation. Studies aimed at validating the questionnaires in other languages have revealed certain weaknesses in these tools. In the case of RSI, it is the scope of the symptoms described, and in the case of RFS it is the lack of clear criteria for describing the changes observed in the larynx [29–32].

The Warsaw Scale had been developed based on the results of studying 249 patients with voice disorders and confirmed reflux disease. Assessment of the morphological image of the larynx is based on a systematic description of the observed changes. The repeatability of abnormalities observed in the larynx and throat has allowed the authors to distinguish 5 degrees of reflux laryngopharyngitis. Grades A and B refer to signs that are widely described and long associated with the occurrence of LPR: inflammation of the mucosa of the anterior pharyngeal commissure and anterior vocal folds, and inflammation of the mucosa of the arytenoid cartilage and of the interarytenoid area. Grades C and D describe lesions in both the larynx and lower throat. They indicate what is happening in the vestibule of the larynx: inflammation and protrusion of the area behind the arytenoid cartilage, vestibular folds, and aryepiglottic folds, sometimes taking the form of a pseudo-tumor of the pharynx. Grade E refers to hypertrophic changes in the larynx, oedemas of the infraglottis, dysfunction of the crico-arytenoid joints, or contact granulomas or overgrowth of the vocal folds.

Here, we have provided an initial justification for using the Warsaw Scale for diagnosing LPR. Our data, obtained from a pilot population of 100 patients, indicate that the scale could be valuable and of clear benefit for clinical practice. Thus, in our population, the scale turned out to be more predictive of LPR (that is, it was more strongly correlated with pH measures) than RFS, RSI, or VHI.

Nonetheless, we must emphasize that our work is exploratory and prospective. Hence, its conclusions should be treated as the basis for further work. There is a need for specific and detailed exploration of how well the Warsaw Scale can effectively diagnose LPR. Although our data show good promise, with the scale substantially outperforming other tools in our population, there are still aspects that need elaboration. Carefully designed studies to assess the scale’s reliability, consistency, and robustness are needed. It will be necessary to replicate our findings in a more heterogenous population in an independent clinical center. That being said, if the reported predictive power were to be confirmed, it would provide ENT practitioners with a highly valuable tool.

The closest tool to the Warsaw Scale is Belafsky's RFS. RFS uses a special sheet on which a single assessment of various morphological changes is made. The Warsaw Scale uses a conjunction of symptoms: to classify the higher stages of LPR, all previous symptoms must be present. RFS takes an experienced doctor about 20 or 30 s, although a less skilled practitioner can take several minutes. For the Warsaw Scale, determining the type and degree of change depends on a videolaryngoscopic examination, after which there is an overall assessment of the image (it does not require individual irregularities to be assessed in terms of their severity). Recently, Lechien has published two papers introducing two new questionnaire tools: the Reflux Symptom Score (RSS) and the Reflux Sign Assessment (RSA). Both questionnaires seek to eliminate the weaknesses of Belafsky's questionnaires [6, 8]. It will be very interesting to check their usefulness in clinical practice and test the LPR predictive ability on a larger population.

### Conclusions

The Warsaw Scale has been demonstrated to be a valuable instrument for the diagnosis of LPR. It provides an alternative approach to assessing the symptoms connected with LPR, and our data suggest it has better performance than any other scale. However, further studies are needed to confirm these findings in other settings, countries, and patient populations.

## Data Availability

The datasets used and/or analysed during the current study are available from the corresponding author on reasonable request.
